# Organizational Culture, Justice, Dehumanization and Affective Commitment in French Employees: A Serial Mediation Model

**DOI:** 10.5964/ejop.8243

**Published:** 2023-08-31

**Authors:** Jean-Félix Hamel, Fabrizio Scrima, Lucie Massot, Benoît Montalan

**Affiliations:** 1 Centre de Recherche sur les Fonctionnements et les Dysfonctionnements Psychologiques (CRFDP, UR7475), University of Rouen Normandy, Rouen, France; 2 University of Paris 8 (IED), Paris, France; Central European University, Vienna, Austria

**Keywords:** commitment, organizational dehumanization, organizational culture, organizational justice, serial mediation

## Abstract

The instrumentality of employees can be considered a common feature of the modern workplace. To investigate the influence of this instrumentalizing culture on organizational performance on the individual level, we tested whether perceived clan values (according to the Competing Values Framework) could explain affective commitment directly and indirectly through perceptions of organizational justice and organizational dehumanization in employees. Using the PROCESS macro, we tested a corresponding serial mediation model in a convenience sample of 306 French employees. Although employees who perceived a lack of clan values were less committed, the observed indirect effect was greater. Our findings highlight the role of perceived organizational culture in influencing affective commitment and how perceived justice and dehumanization may explain part of this relationship. This research also contradicts widespread beliefs stating dehumanizing strategies are universally beneficial in terms of organizational efficiency. Limitations and directions for future research are discussed.

The relationship between organizational culture and various effectiveness criteria has long been a focus of this field of research (Sackman, 2011; Schneider et al., 2013; Wilderom et al., 2000). A meta-analysis by Hartnell et al. (2011) used the Competing Values Framework (CVF) (Cameron & Quinn, 2011) to establish and clarify the culture-performance relationship. The meta-analysis confirmed different types of culture related differently to effectiveness criteria and revealed the dimensions of this widely-used framework (Cameron et al., 2006) provide a better account of overall organizational effectiveness when combined. This finding reinforced the need for organizations to take an interest in their overall *cultural configuration* and its underlying stakes such as occupational health outcomes (Dextras-Gauthier et al., 2012), which explains why it remains the topic of much discussion today (e.g., Kim & Chang, 2019).

Concurrently, the global socioeconomic context promotes a “growing tendency for organizations and leaders to see humans as ‘means’ rather than ‘ends in themselves’”, in stark contradiction with the Kantian moral imperative (Rochford et al., 2017, p. 763). In line with this trend, multiple authors theorized such instrumentalizing practices to have become an ordinary occurrence in the workplace due to widely shared beliefs about their necessity (e.g., Christoff, 2014) or the legitimizing role of neoliberal ideology (Bal & Dóci, 2018). Despite the instrumentalized representation of employees seemingly becoming a fixture of the modern world of work, its influence on employees and their effectiveness in relation to organizational variables remains relatively unknown.

Using the Competing Values Framework (Cameron & Quinn, 2011), the present study aims at investigating whether the extent to which employees perceive their organization to convey humanizing cultural elements (i.e., values) may affect a relevant effectiveness criterion such as affective commitment (Meyer et al., 1989). Our second objective is to test a possible psychological mechanism that could explain, in part, the relationship between perceived organizational culture and affective commitment – i.e., whether it could be mediated by variables such as the perception of organizational justice and the perception of organizational dehumanization. Thus, we hypothesized as to the relationship between all mobilized variables before investigating the corresponding serial mediation model.

## Theoretical Framework

### The Relationship Between Clan Culture and Affective Commitment

The Competing Values Framework frames organizational culture by combining two theoretical axes: *focus* (internal *vs.* external) and *structure* (flexibility *vs.* stability). Crossing them yields four different culture-types (i.e., clan, hierarchy, adhocracy, market) with corresponding—and *conceptually* exclusive—values, foci and desired outcomes for organizations (Cameron & Quinn, 2011). In order to provide empirical evidence that dehumanizing cultural practices may influence organizational effectiveness through individual perceptions, we focused our efforts on the clan culture (*internal* and *flexible*). Clan culture translates an inherent investment in the inner organizational environment and its members (e.g., employees) as well as a belief that “the organization’s trust in and commitment to employees facilitates open communication and employee involvement” (Hartnell et al., 2011, p. 679). In line with the Kantian moral imperative, authors have suggested that the most harmful aspect of instrumentalizing employees may not be considering them as disposable assets, but the act of *only* considering them so (Rochford et al., 2017; Väyrynen & Laari-Salmela, 2018). The core values of clan culture (i.e., human affiliation, attachment) being theoretically antithetical to that of an objectifying psychosocial environment and its main effectiveness criteria including commitment (Hartnell et al., 2011; Schneider et al., 2013), it seemed best suited for our purposes.

The values conveyed by typically clannish organizations gravitate toward positive human interactions (e.g., respect, attachment, support, collaboration) (Schneider et al., 2013). The associated effectiveness criteria of such motivations and means include employee satisfaction and *organizational commitment*—a “psychological state that binds the individual to the organization” (Allen & Meyer, 1990, p. 14). As it describes employees’ emotional attachment to their organization, as well as the extent to which they identify with its *values*, affective commitment should theoretically be closely related to perceived clan culture (Krajcsák, 2018). In fact, Abbott et al. (2005) showed that employees who work in organizations that adopt prosocial values such as humanity are more affectively engaged. In their meta-analysis, Hartnell et al. (2011) identified a strong association between clan culture and affective commitment. Using the same framework, past research suggests employees perceiving their organization to have motivations, means and ends dedicated to their welfare may have a stronger affective bond to it (e.g., Kim, 2014).

H_1_: Perceived clan culture is positively associated with affective commitment.

### Organizational Justice as Mediator

Organizational justice can be considered the integration of multiple research currents each concerned with different aspects of the perception of fairness in the workplace (Greenberg & Colquitt, 2005): *distributive* justice (Adams, 1963; Homans, 1961), *procedural* justice (Thibaut & Walker, 1975) and *interactional* justice (Greenberg, 1993). Values such as trust, tolerance and communication being prominent features of the clan culture, we propose employees that attribute these core values to their organization should perceive procedures, decisions and interactions within the organization as *fair*—i.e., report higher levels of *organizational justice* perceptions.

The influence of culture on justice systems and perceptions has mostly been studied on higher levels (e.g., national, organizational) (James, 2015). Nonetheless, multiple studies successfully explored this relationship on the individual level by investigating the influence of values systems on a variety of employee attitudes including fairness (e.g., Di Stefano et al., 2019; Rita Silva & Caetano, 2014; Schminke et al., 2015; Zhou et al., 2019). In line with these findings, we propose employees may understand un/fairness in their organization through the values they attribute to it (and the motivations and objectives so implied). Simply speaking, it may be easier for an employee to judge interactions in the workplace as un/fair regarding the set of values conveyed by the organization than to interpret these interactions as reflections of organizational goals. For instance, a supervisor being impolite might be attributed to the supervisor’s personality, and does not readily fit in with traditional organizational motives; on the other hand, considering the organization to have little regard for the respect of individuals gives actionable insight into the relative un/fairness of the interaction.

Furthermore, past research theorized supporting employees, communicating with them and ensuring fair processes should lead to high levels of commitment (e.g., Meyer et al., 2002)—a claim substantiated by recent work linking justice perceptions with increased commitment (Jang et al., 2021). Finally, a study by St-Pierre and Holmes (2010) suggested managers were responsible for “implementing and maintaining a culture of trust and justice” (p. 1174), giving organizational justice a mediating role between culture and outcomes such as workplace aggression. We suggest a similar phenomenon could be observed between clan culture and affective commitment: for employees, perceiving their organization to care about them may strengthen their emotional bond directly, but also indirectly via repeated experiences of fair treatment.

H_2_: Perceived clan culture is positively associated with perceived organizational justice (2a), which is positively associated with affective commitment (2b).

H_3_: Perceived organizational justice partially mediates the relationship between perceived clan culture and affective commitment.

### Organizational Dehumanization as Mediator

Numerous currents of research inside and outside of work and organizational psychology (WOP) have taken an interest in the dehumanizing effects of the modern workplace. For instance, dehumanization is a cornerstone of both Marx’s critique of the inherently alienating capitalistic system and Weber’s writings on the impeding bureaucratization of work (see Bell & Khoury, 2011). Related works in recent WOP literature mainly stem from the social psychology of dehumanization (Haslam, 2006) and objectification (Gruenfeld et al., 2008). However, too few of these studies have examined this phenomenon from the perspective of targets (Baldissarri & Andrighetto, 2021; Haslam & Loughnan, 2014), and even less so from that of employees (or in the context of work at large). This shortcoming in the literature has notably been addressed by contextualizing *metadehumanization*—which describes the subjective experience of feeling dehumanized by others (Kteily et al., 2016)—to the work domain.

Thus, exploring instrumentalization from the perspective of employees and recognizing that it may also emanate from the organization itself (see Väyrynen & Laari-Salmela, 2018), Bell and Khoury (2011) defined *organizational dehumanization* as “the experience of an employee who feels objectified by his or her organization, denied personal subjectivity, and made to feel like a tool or instrument for the organization’s ends” (p. 170). In other words, organizational dehumanization represents the extent to which employees feel their organization dehumanizes and objectifies them for its own benefit. As such, it examines the role of higher-level phenomena than objectifying *interpersonal* interactions, such as reifying human resources management practices (see Shields & Grant, 2010, cited by Baldissarri & Andrighetto, 2021, p. 86). A burgeoning literature has since identified organizational dehumanization as a frequent mediator between critical constructs of WOP research such as organizational justice and turnover intentions (Bell & Khoury, 2016) or abusive supervision and affective commitment (Caesens et al., 2019).

Recent work suggests organizational dehumanization perceptions may arise when individuals feel hindered in meeting their psychological needs (i.e., competence, autonomy and control) (Demoulin et al., 2021). Thus, organizational dehumanization also speaks to the representation employees have formed of their employer-employee relationship insofar as it contributes to their needs not being met. In this study, we propose this representation may be informed by cultural elements such as the perceived organizational values underpinning the observable behaviors conveying an instrumentalized representation of employees. While dehumanizing strategies may be common in modern organizational settings (Christoff, 2014), organizations which exhibit clan values tend to establish conditions aiding employees in maintaining feelings of competence, self-esteem and support (Dextras-Gauthier et al., 2012; Hartnell et al., 2011). If conditions help employees fulfill this psychological need, there is reason to think it might prevent organizational dehumanization from emerging in the first place (Demoulin et al., 2021). Simply speaking, employees perceiving their organization to foster a culture which considers their psychological needs should not feel considered as *mere instruments* devoted to its ends. Finally, given both the already established negative relationship between organizational justice and organizational dehumanization (e.g., Bell & Khoury, 2016) and the one hypothesized above (*H_2a_*), we expect organizational justice to play the role of mediator between perceived clan culture and organizational dehumanization—i.e., clan culture may influence organizational dehumanization directly but also indirectly by influencing un/fairness perceptions.

H_4_: Perceived clan culture is negatively associated with feelings of organizational dehumanization (4a). Perceived organizational justice partially mediates the relationship between perceived clan culture and organizational dehumanization (4b).

**Figure 1:**
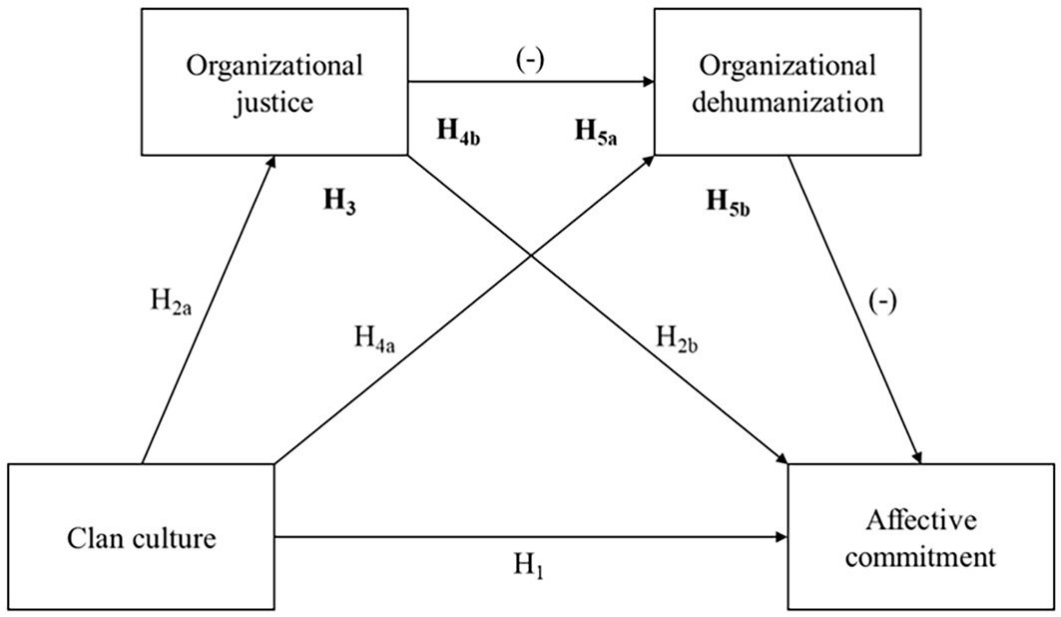
Hypothesized Serial Mediation Model

In their seminal work, Bell and Khoury (2011) suggested feelings of organizational dehumanization would diminish employees’ attachment and encourage them to dissociate from their organization both physically and emotionally. This would notably translate into a decrease in organizational commitment, which other work has since supported (e.g., Caesens et al., 2019; Stinglhamber et al., 2021). The mediating role of organizational dehumanization in the relationship between organizational justice and turnover intentions has also been reported (Bell & Khoury, 2016). We propose the same can be said of the justice-commitment relationship: un/fair interactions in the workplace might affect the psychological bond between and employee and their organization directly, but also indirectly by fostering a detrimental representation of the employer-employee relationship in the latter’s eyes. The sum of all our hypotheses amounts to a serial mediation model represented in Figure 1 (mediation hypotheses are in bold).

H_5_: Organizational dehumanization partially mediates the relationship between perceived organizational justice and affective commitment (5a) as well as perceived clan culture and affective commitment (5b).

## Method

### Sample and Procedure

Participants (*N* = 306) were recruited online, via email and social media. Prospecting participants were required to report reading French and being employed in order to participate. First, they were informed about the general purpose of the study and assured that their anonymity would be guaranteed. Finally, they were asked to give their consent to participate before filling the survey. Almost two-thirds of participants were women (65%). The mean age of participants was 41 years old (*M* = 40.63, *SD* = 9.59).

### Instruments and Statistical Analyses

Statistical analyses were conducted using SPSS Statistics 25, Amos Graphics 24 and the 4.2 version of the PROCESS macro (Hayes, 2017). Among other advantages, PROCESS allows for the isolation of each mediator’s indirect effect and addresses some of the issues traditionally associated with the popular approach described by Baron and Kenny (1986) or the Sobel test (see Cole et al., 2008). Internal consistency was determined using McDonald’s omega, which is considered by some authors to be more robust than Cronbach’s alpha (e.g., Hayes & Coutts, 2020).

#### Perceived Clan Culture

To assess perceived clan culture, we used a dimension of the Organizational Culture Profile (OCP, O’Reilly et al., 1991) comprised of 7 values theoretically associated with this form of organizational culture (Cameron & Quinn, 2011) (e.g., respect for individuals’ rights, fairness, being people-oriented). Participants had to rate on a 1 (*not well at all*) to 5 (*very well*) Likert-scale the extent to which each value described the organization they are employed by (ω = .90). The OCP is recommended in the quantitative study of organizational culture and its influence on occupational health outcomes (Dextras-Gauthier et al., 2012). In 2013, Marchand et al. (2013) demonstrated their French version could be used to accurately replicate the framework’s culture-types in this context, including clan culture. A confirmatory factor analysis suggests a satisfactory fit of the measurement model (χ^2^ = 32.84, *df* = 12, *p* < .01, χ^2^/*df* = 2.73, CFI = .98, NNFI = .97, SRMR = .03).

#### Organizational Dehumanization

Organizational dehumanization was measured using the scale created and validated by Caesens et al. (2017). A French version was provided by the authors. The scale is composed of 11 statements (e.g., “My organization considers me as a tool to use for its own ends”, “My organization treats me as if I were an object”) regarding how workers think they are treated by their organization as a whole. Answers were self-reported on a 1 (*strongly disagree*) to 7 (*strongly agree*) Likert-scale. Internal consistency was very high (ω = .95). The results of a confirmatory factor analysis indicate a good fit to the data (χ^2^ = 102.14, *df* = 37, *p* < .001, χ^2^/*df* = 2.76, CFI = .98, NNFI = .97, SRMR = .03).

#### Organizational Justice

Validated by Jouglard-Tritschler and Steiner (2005) and adapted to the French organizational setting by Piasecki (2017), Colquitt’s (2001) scale was used to measure organizational justice. The scale’s four dimensions—distributive, procedural, interpersonal and informational justice—were respectively represented by 4, 7, 4 and 5 items. They were formulated as questions such as “Does your outcome reflect what you have contributed to the organization?” or “Does your supervisor treat you in a polite manner?” For each query, participants had to answer on a 1 (*not at all*) to 5 (*absolutely*) Likert-scale. For the purpose of analyzing the construct as a whole in our serial mediation model, we averaged the scores of all dimensions into a global organizational justice score (ω = .95). Confirmatory factor analysis suggests the measurement model fits well to the data (χ^2^ = 485.24, *df* = 166, *p* < .001, χ^2^/*df* = 2.92, CFI = .94, NNFI = .93, SRMR = .05).

#### Affective Commitment

Affective commitment was measured using the French version (Belghiti-Mahut & Briole, 2004) of the Organizational Commitment Scale (Meyer et al., 1993). The affective dimension of the scale contains 6 items for which participants had to answer on a 5-point Likert-scale (1 = *totally disagree* and 5 = *totally agree*). Items include statements such as “I would be very happy to spend the rest of my career with this organization” or “This organization has a great deal of personal meaning for me” (ω = .83). Confirmatory factor analysis suggests a good fit of the measurement model (χ^2^ = 13.68, *df* = 5, *p* < .05, χ^2^/*df* = 2.74, CFI = .98, NNFI = .96, SRMR = .03).

#### Control Variables

Control variables were chosen based on their potential relationships with dependent variables according to previous literature. For instance, Bell and Khoury (2016) showed gender could moderate the effect of procedural justice on feelings of organizational dehumanization. Tenure was previously shown to correlate with affective commitment (Meyer et al., 2002). Tenure was measured by asking participants to place themselves regarding six tenure intervals (less than one year, followed by 5-years increments up to 21 years and more). Control variables were then analyzed according to the recommendations of Becker et al. (2016). Standardized coefficients from hierarchical regression models were compared with and without the relevant control variables. In all cases except for tenure, the differences could be considered negligible according to these authors’ decision rule (Δβ < .1). Hence, tenure was included as a covariate in further analyses.

## Results

### Preliminary Analysis

Considering the cross-sectional design of this study, we tested whether the data were influenced by the common-method bias (Podsakoff et al., 2012) using structural equation modeling. We compared four alternative models. The first was unifactorial—all the items of the four scales loaded on a single latent factor. The second model was composed of a latent factor on which clan culture items loaded and another covaried latent factor on which all other items did. In the third model, items related to affective commitment and organizational justice loaded on a common covaried latent factor while the other items still loaded on their respective ones. The final model contained four covaried factors (clan culture, organizational justice, organizational dehumanization and affective commitment) in which each item loaded on its respective latent factor.

We calculated the following fit indices for each model: the Carmines-McIver index (i.e., the ratio between χ^2^ and degrees of freedom), the value of which must be between 2 and 3 to indicate a satisfactory fit (McIver & Carmines, 1981); the Comparative Fit Index (CFI) and the Non-Normed Fit Index (NNFI), with values above .90 considered to reflect good fit given our sample size (e.g., Hair et al., 2014); the Standardized Root Mean Square Residual (SRMR), with values lower than .08 considered as satisfactory (Hu & Bentler, 1999). The models were also compared using Δχ*^2^*. We used the maximum likelihood method to estimate parameters. The results of this procedure are provided in Table 1. Only the last model managed to satisfy all retained fit criteria; it is also confirmed to be the best model by Δχ*^2^* tests. Overall, these results suggest our data are not subject to common-method bias.

**Table 1:** Common-Method Bias Analysis

Model	χ*^2^*	*df*	*p*	χ*^2^/df*	CFI	NNFI	SRMR	Δχ*^2^*	Δ*df*	*p*
Model 1 – single factor	2293	346	***	6.63	.70	.67	.10			
Model 2 – covaried 2 factors	1722	345	***	4.99	.79	.77	.10	571	1	***
Model 3 – covaried 3 factors	1188	343	***	3.46	.87	.86	.07	534	2	***
Model 4 – covaried 4 factors	891	340	***	2.62	.92	.91	.06	297	3	***

*Note*. *N* = 306. *df* = degrees of freedom. χ*^2^/df* = Carmines-McIver Index. CFI = Comparative Fit Index. NNFI = Non-Normed Fit Index. SRMR = Standardized Root Mean Square Residual.

***p < .001.

### Descriptive Statistics

Descriptive statistics, scale reliability as well as correlations between variables were reported in Table 2. All skewness and kurtosis values were between -1 and 1, suggesting small departures from normality. Organizational dehumanization was significantly (*p* < .001) and negatively related to perceived clan culture (*r* = -.57), affective commitment (*r* = -.47) as well as perceived organizational justice (*r* = -.65). As expected, perceived clan culture was positively and significantly (*p* < .001) associated with affective commitment (*r* = .50), as well perceived organizational justice (*r* = .63). Finally, affective commitment was positively and significantly (*p* < .001) related to perceived organizational justice (*r* = .51).

**Table 2:** Descriptive Statistics and Bivariate Correlations

Variable	*M*	*SD*	S	K	1	2	3	4	5	6
1. Clan culture	3.24	0.86	-.25	-.32	*(.90)*					
2. Organizational Justice	3.26	0.86	-.34	-.49	.63***	*(.95)*				
3. Organizational Dehumanization	3.77	1.58	.11	-.82	-.57***	-.65***	*(.95)*			
4. Affective Commitment	3.22	0.89	-.43	.08	.50***	.51***	-.47***	*(.83)*		
5. Sex					-.06	-.04	.08	-.09		
6. Age	40.63	9.59	.13	-.55	.03	-.07	-.01	.07	-.19**	
7. Tenure					.01	-.02	.09	.14*	-.25***	.61***

*Note*. *N* = 306. Sex (0 = male, 1 = female). Tenure (0 = less than one year – 6 = 21 years or more). S = Skewness. K = Kurtosis. Diagonal: McDonald's ω.

*p < .05. **p < .01. ***p < .001.

### Hypothesis Tests

Table 3 summarizes all of the models tested in the serial mediation analysis. All models were significant (*p* < .001). Based on 10,000 bootstrap samples with 306 cases, none of the 95% confidence intervals contained zero except for constants. As expected (H_1_), the total effect of perceived clan culture on affective commitment was positive and significant (β = .491, SE = .049, *p* < .001). Our hypotheses were also supported by the predictor’s effects on perceived organizational justice (H_2a_) and organizational dehumanization (H_4a_). H_2b_ was supported by the positive and significant effect of perceived organizational justice on affective commitment. Our results also seem consistent with past literature regarding the relationship between perceived organizational justice and organizational dehumanization as well as organizational dehumanization and affective commitment. Results indicate tenure had a small positive effect on both organizational dehumanization and affective commitment.

**Table 3:** Serial Mediation Results

			95% CI		
Predictor	β	*SE*	LL	UL	*p*
M1 (Organizational justice)					
X	.63	.05	.55	.72	***
M1					
M2					
Constant	.01	.04	-.09	.09	n.s.
Tenure	-.02	.05	-.11	.06	n.s.
*R^2^* = .40					
*F*_(2, 303)_ = 101.19***					
M2 (Organizational dehumanization)					
X	-.26	.05	-.37	-.16	***
M1	-.48	.05	-.59	-.38	***
M2	—	—	—	—	—
Constant	.01	.04	-.08	.08	n.s.
Tenure	.09	.04	.01	.17	*
*R^2^* = .47					
*F*_(3, 302)_ = 88.86***					
Y (Affective commitment)					
X	.22	.06	.10	.34	***
M1	.24	.07	.11	.38	***
M2	-.20	.06	-.33	-.08	**
Constant	.01	.05	-.09	.09	n.s.
Tenure	.16	.05	.07	.26	***
*R^2^* = .35					
*F*_(4, 301)_ = 41.20***					

*Note*. *N* = 306. All variables were standardized. X = Clan culture. CI = confidence interval. LL = lower limit. UL = upper limit. Confidence intervals were determined using 10,000 bootstrap samples.

*p < .05. **p < .01. ***p < .001.

**Figure 2:**
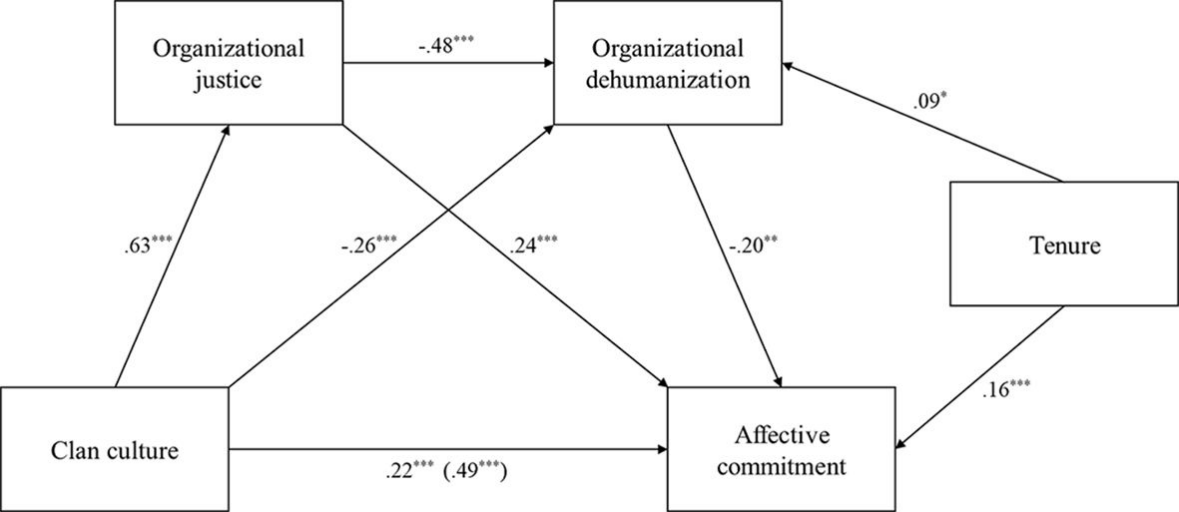
Serial Mediation Model of Clan Culture’s Effect on Affective Commitment

Indirect effects and their bootstrapped 95% confidence intervals are reported in Table 4. None contain zero, which supports all remaining mediation hypotheses (H_3_, H_4b_, H_5a_ and H_5b_). The largest effect is the one translating perceived clan culture’s indirect influence on affective commitment through perceived organizational justice. Smaller indirect effects can also be observed through organizational dehumanization mediating this relationship or by considering both mediators. Finally, the total indirect effect (β = .269) was larger than the direct effect (β = .222). A visual representation of the overall model has been provided (Figure 2).

**Table 4:** Bootstrapped Indirect Effects of Perceived Clan Culture on Affective Commitment

			Boot. 95% CI	
Path	*β*	*Boot. SE*	LL	UL
PCC → OJ → AC	.154	.042	.077	.237
PCC → OD → AC	.054	.020	.019	.096
PCC → OJ → OD → AC	.062	.020	.024	.104
Total effect	.491	.049	.394	.588
Direct effect	.222	.062	.100	.344
Total indirect effect	.269	.043	.186	.356

*Note*. *N* = 306. All variables were standardized. PCC = perceived clan culture. OJ = organizational justice. OD = organizational dehumanization. AC = affective commitment. CI = confidence interval. LL = lower limit. UL = upper limit. Confidence intervals were determined using 10,000 bootstrap samples.

## Discussion

While the relationship between organizational culture and effectiveness criteria has been the object of a large body of work in the past twenty years, few studies have investigated it on the individual level. In the larger context of employees sometimes feeling considered as *mere instruments* by organizations, the purpose of our study was twofold. First, we tested whether a traditionally studied effectiveness criterion in the form of affective commitment (Meyer et al., 1989) could be influenced by the manner in which employees perceive organizational culture; more specifically, whether the extent to which employees perceived values associated with the clan culture—often considered *humanizing* values—could explain their level of affective commitment. Second, we investigated this effect in relation to two potential mediators—un/fair treatment (i.e., perceived organizational justice) and feeling considered as *means to the organization’s ends* (i.e., organizational dehumanization). Taking past literature into consideration, we proposed a serial mediation model which integrated already established relationships between these variables to our hypotheses.

All hypotheses were supported by the data. All other things being equal, employees who perceive their organization to lack in their enforcement of *human*-oriented values (e.g., tolerance, trust, support) tend to be less committed in the first place. However, it is of note that the overall indirect effect was greater than the direct effect: this *affective distancing* might not only find its source in this initial perception but also in its reverberation through other variables. This perceived lack of humanizing elements might simultaneously make it harder for employees to find their organizational environment fair yet easier for them to believe they are not being considered in a morally appropriate way—as whole human beings and not simply as *human resources*. In doing so, it might encourage employees to question their employer-employee relationship in a more profound way, which begs a simple question: how long until they’re no longer content with feeling treated this way? Indeed, as these variables may for instance foster higher turnover intentions (e.g., Lagios et al., in press; Stanley et al., 2013) or emotional exhaustion (Caesens & Stinglhamber, 2019), it would behoove organizations to not only consider culture from a *macro* perspective, but also from an individual one. As all culture-types collaborate with one another to form a holistic organizational pattern (Hartnell et al., 2011) even organizations not traditionally centered on clan values should take heed of such results.

Organizational culture has long been considered a multilayered construct (e.g., Schein, 2010). Part of the confusion surrounding its study revolves around scholars not acknowledging its different levels of analysis or not distinguishing it from organizational climate (Ostroff et al., 2013). To these points, the provided results are not intended for the analysis of a specific organizational unit but rather as evidence of employees’ active role in influencing the outcomes of organizational values. Consequently, and to the second point, our operationalization of perceived organizational culture can be understood as the individual integration of both publicly embraced organizational values (*corporate* culture) and the ones employees feel are actually conveyed by their everyday experience of the work environment (i.e., climate).

In their correlational study, Auzoult and Personnaz (2016) introduced the idea that organizational culture may play a role in employee self-objectification (i.e., internalizing an objectified representation of the self). In a similar fashion, recent work using both cross-sectional and experimental data proposed the existence of a causal relationship between instrumentalizing treatment and self-objectifying perceptions (Baldissarri & Andrighetto, 2021; Sainz & Baldissarri, 2021). These studies questioned the representation employees had of *interpersonal* relationships (e.g., with their supervisors, with a colleague). However—as highlighted by multiple scholars (e.g., Eisenberger et al., 2002; Ng & Sorensen, 2008)—they cannot be considered the same as the relationship between employees and their organization as an entity. Building on this literature, our study provides novel empirical evidence suggesting an individual’s perception of their organization’s culture may influence how they feel considered by it *as a whole* in terms of fairness and organizational dehumanization. This implies employees are not passively influenced by their organization’s culture—in fact, rather the contrary: by appropriating the underlying values and interpreting their relative detrimental or beneficial role in promoting fairness or helping them fulfil their needs, employees appear to *actively* participate in shaping the effect of culture on their own effectiveness.

### Limitations and Future Directions

Of course, these conclusions need to be put into perspective, as they exclusively relied on self-reported and cross-sectional data. Although we provided statistical evidence that our data was not subject to common-method bias (Podsakoff et al., 2012), our research design is unable to support causal relationships between the mobilized variables. Consequently, our choice of predictor may be questioned; conversely to our model, one could argue justice perceptions may inform values perceived by employees (i.e., reversed directionality). Such choices should preferably rely on theoretical arguments rather than statistical ones (Hair et al., 2014). Therefore, we acknowledge the relationship between clan culture and organizational justice perceptions may be circular and recursive—much like Bell and Khoury (2011) admitted the one between organizational justice and organizational dehumanization might be. Additionally, cross-sectional data traditionally does not allow for robust conclusions on mediation hypotheses (see Maxwell & Cole, 2007). Hence, future research should turn to both longitudinal and experimental designs so as to provide more robust conclusions regarding the directionality of the proposed paths.

Alongside the addition of pertinent variables further specifying which employees could be the most affected by perceived clan values (part-time *vs.* full-time; smaller *vs.* larger organizations, etc.), qualitative studies could also shed new light on the underlying processes. In addition, the validity of our findings would benefit from being reproduced in the context of single organizational units as well as being included in a multilevel analysis of the culture-effectiveness relationship. In doing so, future work would not only confirm the tendency we uncovered in this paper persists within the confines of distinct organizations (or their subparts) but also integrate these findings to already documented culture-effectiveness models.

Moreover, while the size of our sample was satisfactory, it is possible to question its representativeness of employees in general. For instance, almost 8 out of 10 participants reported being full-time employees (79%) and a majority of participants had the equivalent of a Master’s degree or an even higher form of education (61%). Although no specific effect was detected in relation to these variables, future studies may want to investigate their role as potential moderators. Overall, they should use more representative samples so as to extend any replicated findings to the general population. Finally, using tenure intervals to assess its influence on our dependent variables not being optimal, future work should measure it continuously.

### Conclusion

The first objective of this study was to investigate the relationship between employee perceptions of organizational values conveying interest in fostering a fulfilling work environment (i.e., clan culture) and affective commitment. We also explored the mediating role of the extent to which employees feel fairly treated by organizational agents (i.e., organizational justice) and their organization as a whole (i.e., organizational dehumanization) between these variables. Consistent with previous literature (Bell & Khoury, 2016; Caesens et al., 2019; Jang et al., 2021), our results outline a theoretically and practically relevant serial mediation model which could be the basis of new research avenues or psychosocial interventions. While they reinforce the clan—commitment relationship, organizational justice and organizational dehumanization perceptions relate to different work relationships (i.e., interpersonal *vs*. employer-employee). It may be worthwhile to consider the role of managers’ organizational embodiment (Eisenberger et al., 2010) by testing whether organizations conveying low clan values may gain by having managers *compensate* (and vice-versa). Practically, our findings should encourage organizations to veer away from the pervasive instrumental representation of employees they might carry. They can also be appropriated by managers willing to recognize that making employees’ intrinsic value (i.e., both *means* and *ends in themselves*) one of their core preoccupations may positively shape the reverberations of *top*-*down* organizational phenomena on employee effectiveness.

## Data Availability

The data used and analyzed in this study are available from the corresponding author upon reasonable request.
